# Hypnotherapy or transcendental meditation versus progressive muscle relaxation exercises in the treatment of children with primary headaches: a multi-centre, pragmatic, randomised clinical study

**DOI:** 10.1007/s00431-018-3270-3

**Published:** 2018-10-24

**Authors:** M. C. Jong, I. Boers, H. A. van Wietmarschen, E. Tromp, J. O. Busari, R. Wennekes, I. Snoeck, J. Bekhof, A. M. Vlieger

**Affiliations:** 1 Louis Bolk Institute, Kosterijland 3-5, 3981 AJ Bunnik, The Netherlands; 2 Mid Sweden University, Sundsvall, Sweden; 3 St. Antonius Ziekenhuis, Nieuwegein, The Netherlands; 4 Zuyderland Medisch Centrum, Heerlen, the Netherlands; 5 Isala, Zwolle, The Netherlands

**Keywords:** Hypnotherapy, Meditation, Headache, Relaxation, Pain, Children

## Abstract

Many children suffer from headaches. Since stress may trigger headaches, effective techniques to cope with stress are needed. We investigated the effectiveness of two mind-body techniques, transcendental meditation (TM) or hypnotherapy (HT), and compared them with progressive muscle relaxation (PMR) exercises (active control group). Children (9–18 years) suffering from primary headaches more than two times per month received either TM (*N* = 42), HT (*N* = 45) or PMR (*N* = 44) for 3 months. Primary outcomes were frequency of headaches and ≥ 50% reduction in headaches at 3 and 9 months. Secondary outcomes were adequate relief, pain coping, anxiety and depressive symptoms, somatisation and safety of treatment. Groups were comparable at baseline. Headache frequency was significantly reduced in all groups from 18.9 days per month to 12.5 and 10.5 at respectively 3 and 9 months (*p* < 0.001), with no significant differences between the groups. Clinically relevant headache reduction (≥ 50%) was observed in 41% and 47% of children at 3 and 9 months respectively, with no significant differences between the groups. No differences were observed in secondary outcome measures between the intervention groups. No adverse events were reported.

*Conclusion*: All three techniques reduced primary headache in children and appeared to be safe.

*Trial registration*: NTR 2955, 28 June 2011 (www.trialregister.nl)

**Table:** 

**What is Known:** • *Stress may be an important trigger for both tension type headache and migraine in children.* *• Good data are lacking on the effect of transcendental meditation, hypnotherapy or progressive muscle relaxation as possible stress-reducing therapies in children with primary headaches.*	
**What is New:** *• Three non-pharmacological techniques,* i.e., *transcendental meditation, hypnotherapy and progressive muscle relaxation exercises, all result in a clinically significant reduction of headaches and use of pain medication.* *• No large differences between the three techniques were found, suggesting that children can choose either one of the three techniques based on personal preferences.*

## Introduction

Primary headache has a high prevalence in children. Worldwide, more than 50% of children and adolescents suffer from recurrent headaches, with tension-type headache (TTH) and migraine as the most prevalent headache syndromes [37]. Both TTH and migraine can have a significant impact on children’s quality of life and emotional state, often continuing into adulthood [8]. It may result in missed school days, disturbed peer and family relationships and emotional stress [30].

Treatment of primary headache can prove difficult; although many different medications are used in the acute treatment, only triptans and ibuprofen have some proven effect on pain reduction, but both are associated with adverse events such as fatigue, dizziness, dry mouth, nausea or vomiting, and can even result in headaches due to medication overuse [25, 33]. A recent study showed that the commonly used preventive medications, amitriptyline and topiramate, were as effective as placebo in the reduction of the number of headaches and had higher rates of adverse events [23]. There is good evidence that cognitive behavioural therapy (CBT) can reduce the frequency and severity of headaches in children [21]. In practice, however, CBT is not always feasible, since psychological treatment may not be accepted by patients, CBT can be costly and time-intensive, and may only be effective in older children, capable of metacognition [11, 16].

Another therapeutic approach is the use of mind-body techniques aiming at stress reduction, since stress is viewed as an important headache trigger, both in TTH and migraine [12, 26, 29]. Stress management programs can improve headache severity and frequency compared with no stress management, but the quality of evidence is low [2, 31]. Mind-body techniques such as meditation, biofeedback and hypnosis may reduce stress, but it is unknown if they differ in effectiveness. One study showed that spiritual meditation resulted in a greater decrease in the frequency of headaches than secular meditation or muscle relaxation [35]. In another study, biofeedback + relaxation was more effective than relaxation alone [20]. Both studies however were performed in adults. Data on comparative effectiveness of mind-body techniques in children are lacking. It was therefore decided to investigate the effectiveness of two very different mind-body techniques that have shown a positive effect on pain, stress and anxiety, i.e., transcendental meditation (TM) and hypnotherapy (HT).

It has been reported that TM decreases physiological indices of stress [7] and reduces anxiety [9]. However, no study has specifically investigated the effect of TM on headache in paediatric patients. It has been reported that hypnotherapy is also an effective method for reduction of chronic pain, stress and anxiety [10, 19, 27, 28]. One observational study reported that HT in children may positively contribute to the reduction of headache complaints, with a lasting effect, but good randomised clinical trials (RCTs) are lacking [14].

Thus, both TM and HT seem promising non-pharmacological treatment options in the treatment of children with primary headaches. The aim of this pragmatic, randomised controlled study was therefore to compare the effectiveness of two mind-body techniques, e.g., TM or HT, with progressive muscle relaxation (PMR) exercises on paediatric primary headache. All three interventions were added to standard medical treatment.

## Materials and methods

### Study design

This pragmatic, randomised controlled clinical trial with three parallel groups was executed in six hospitals in the Netherlands: the St. Antonius Ziekenhuis (Nieuwegein), Canisius Wilhelmina Ziekenhuis (Nijmegen), the Atrium Medisch Centrum Parkstad (Heerlen), Isala (Zwolle), Maxima Medisch Centrum (Veldhoven) and the Juliana Kinderziekenhuis (the Hague). Medical ethical approval was obtained from the METOPP Tilburg (Study Number NL 37155.28.11; M416).

### Study population

All children between 9 and 18 years, suffering from primary headache at least two times per month, with the ability to understand and speak the Dutch language, and with access to phone and internet were eligible for inclusion. Children with secondary causes for headache were excluded from the study. Further exclusion criteria were epilepsy, other serious neurological diseases and previous treatment with hypnotherapy, meditation or PMR. Written informed consent by the child and both parents were obtained prior to inclusion and any examinations or measures related to the study.

### Intervention

All children in the study were provided with standard medical treatment according to the hospital guidelines. This was an individual mixture of symptomatic (pain, antiemetic) and prophylactic medication. It included attention to daily regimen: sleep hygiene, diet, caffeine and stress reduction. Children were randomised to either the TM group, the HT group or the PMR exercises (active control group). The technique of TM was taught by qualified trainers in a standardised course program as described by Tanner et al. [32]. After the introduction meeting with the parents, the child was taught and directed in the meditation techniques and exercises during five individual sessions of 60 to 90 min. Children were instructed to practise TM two times a day for 10 min over a period of 3 months.

Children in the HT group received six individual HT sessions of 50 min under supervision of a hypnotherapist over a period of 3 months. The HT protocol used in this study was an adaptation of the Dutch hypnosis protocol for paediatric abdominal pain [34]. After every session, children were asked to practise self-hypnosis or listen to the recorded sessions at home, at least once a day.

Children in the control group were offered PMR exercise sessions, including home exercises (once a day), taught by an experienced physiotherapist or psychologist with a maximum of six times within the 3-month intervention period.

### Randomisation

Patients were stratified per hospital into six groups and subsequently randomised using separate randomisation lists as generated by the computerised Random Allocation Software Program employing a random block size of six to guarantee balanced allocation. The study investigator was blinded for allocation sequence and assigned subjects to interventions.

### Outcome measures

Demographics and clinical characteristics were measured at baseline. Headache severity at baseline was measured on a four-point Likert scale, ranging from light to severe headache. Outcomes were measured at baseline, and at 3 and 9-month follow-up. The primary outcome measure was the mean frequency of primary headache attacks per 4 weeks, registered by the patient in a 4-week headache diary [4]. A reduction of > 50% in the mean frequency of primary headache attacks was considered clinically relevant and therefore analysed as a second primary outcome measure.

For clinical studies on chronic pain, it has been recommended to also investigate other possible pain-related outcome parameters such as coping strategies and change of behaviour and affective state parameters [22]. Therefore, besides the headache frequency, also adequate relief, anxiety and depression symptoms, coping strategies and somatisation scores were measured as secondary outcome measures. Changes in anxiety and depression scores were measured by the 25-item Revised Children’s Anxiety and Depression Scale-short version (RCADS-25) [5], changes in coping mechanisms by the 39-item Dutch Pain Coping Questionnaire (PCQ) [24] and changes in somatisation by the 35-item Children Somatisation Inventory (CSI) [18]. In addition, the occurrence of intervention-related adverse events was monitored, treatment preference at the start of the study and use of pain medication.

### Sample size

Sample size calculation was based on the assumption that after 3 months of HT and TM, 40% of the children would report a 50% or more reduction in mean frequency of headache, compared to 12% of the children in a standard medical treatment group only. This assumption was based on previous studies on the effect of relaxation therapies in children with primary headache [15], demonstrating a 30–60% responder rate dependent on the relaxation technique versus baseline. The calculated sample size was 111 children, 37 in each group. To allow for an estimated 20% drop-out, the total number of children per group was set at 47, which made 141 children in total. The sample size was calculated with an 80% power and a 0.05 significance level.

### Statistical analysis

Statistical analyses were performed according to the full analysis set (FAS; including all children that were randomised into the study), intention-to-treat principle (ITT; including all children who were randomised and had at least completed one follow-up questionnaire), as well as according to the “per-protocol” (PP) principle (including all children who had executed the intervention as described in the protocol). All statistical analyses were performed on both the ITT and PP datasets, except for the baseline characteristics and adverse events which were performed on the FAS dataset. Missing data was replaced with the last available observation for that variable according to the last observation carried forward (LOCF) approach. Treatment effects at 3 months and at 9 months were analysed with paired *t* tests for continuous variables and McNemar’s tests for nominal variables [17] for each group separately. Differences between the intervention groups were analysed with one-sided ANOVAs for continuous variables and chi-squared tests for nominal variables. Pearson correlations between headache frequency differences of baseline and 9 months and baseline scores on the RCADS, CSI, PCQ and the number of times that the children applied the intervention were calculated. Sub-group analyses were performed for gender, age and type of headache. Data were analysed using SPSS (version 21.0).

## Results

### Study population

**Fig. 1:**
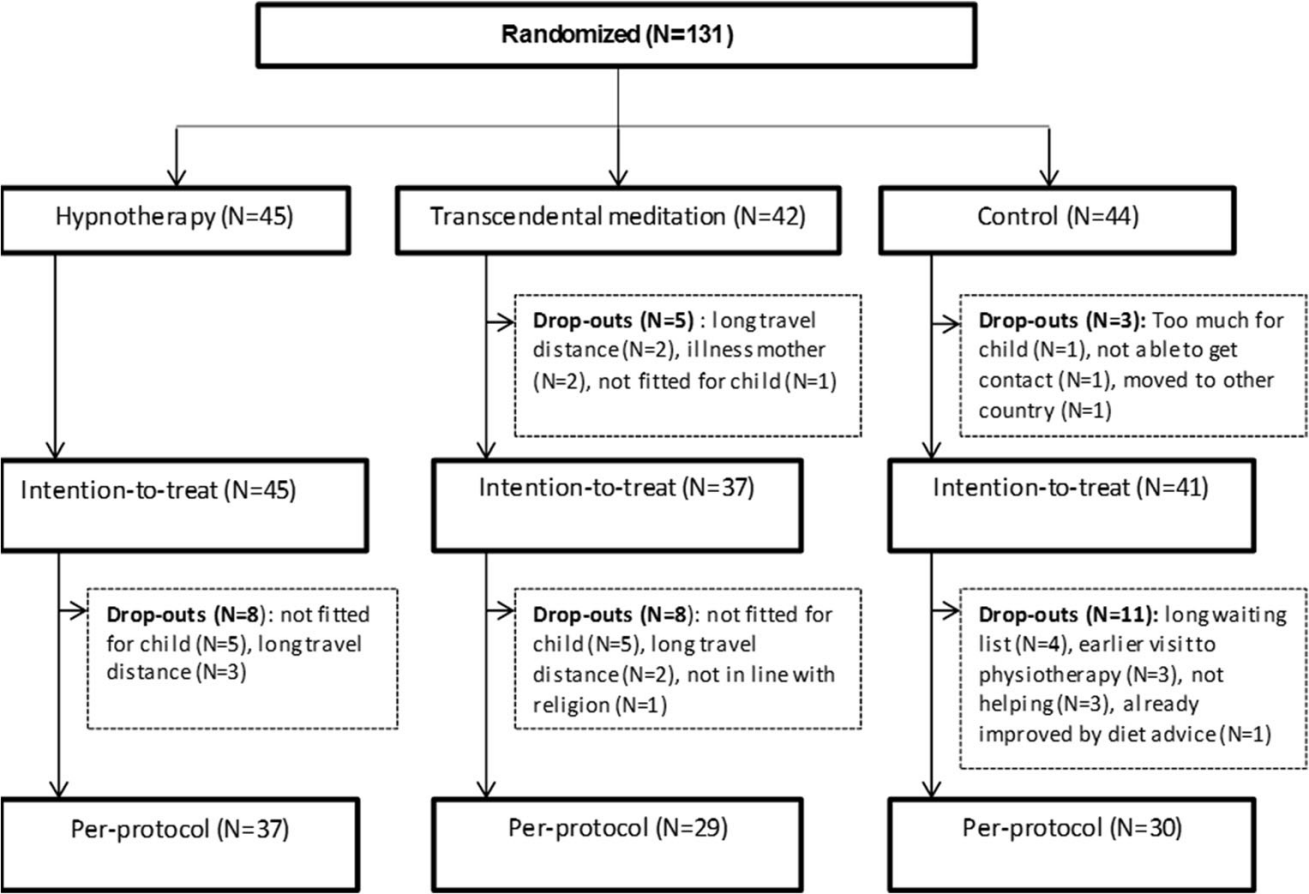
Flow diagram of children in the study

A total of 131 children were randomised into the study in the period from May 2012 to August 2016: 45 children in the HT group, 42 in the TM group and 44 children in the PMR group. As shown in Fig. 1, eight children dropped out before the start of the study. Of these drop-outs, no follow-up data were available. A total of 123 children were included in the ITT analysis: 45 children in the HT group, 37 in the TM group and 41 in the PMR group. During the intervention period of the study, another total of 27 children dropped out (8 in HT, 8 in TM, 11 in PMR). Therefore, the PP analysis was performed in a total number of 96 children: 37 children in the HT group, 29 children in the TM group and 30 children in the PMR group (Fig. 1).

In Table 1, demographic and clinical characteristics are shown of the three groups at baseline. The mean age of participants was 13.3 years. Most children were female (77%), and the most frequently reported type of headache was tension-type headache (60.3%). The mean frequency of primary headache attacks per 4 weeks was 18.4 (SD 9.4). No significant differences at baseline were found between the three intervention groups (Table 1).

**Table 1:** Demographic and clinical characteristics of children in the study

Characteristics	Value	All (N = 131)	HT (*N* = 45)	TM (*N* = 42)	PMR (*N* = 44)
Age (year)	Average age ± SD	13.3 ± 2.5	13.0 ± 2.3	13.9 ± 2.4	12.9 ± 2.7
Gender, *N* (%)	Boys	30 (23%)	7 (16%)	9 (21%)	14 (32%)
	Girls	101 (77%)	38 (84%)	33 (79%)	30 (68%)
Hospital, *N* (%)	Atrium	23 (18%)	8 (18%)	7 (17%)	8 (18%)
	CWZ	9 (7%)	3 (7%)	3 (7%)	3 (7%)
	Antonius	27 (21%)	10 (22%)	9 (21%)	8 (18%)
	Isala	46 (35%)	14 (31%)	15 (36%)	17 (39%)
	MMC	6 (5%)	3 (7%)	1 (2%)	2 (5%)
	JKZ	20 (15%)	7 (16%)	7 (17%)	6 (14%)
Diagnosis, *N* (%)	Migraine	21 (16%)	7 (16%)	7 (17%)	7 (16%)
	Tension-type headache	79 (60%)	30 (67%)	24 (55%)	26 (59%)
	Migraine/tension type	23 (18%)	6 (13%)	8 (19%)	9 (21%)
	Chronic daily headache	7 (5%)	2 (4%)	3 (7%)	2 (5%)
	Cluster headache	1 (1%)	–	1 (2%)	–
Headache duration	Years, average score ± SD	2.8 ± 2.9	3.0 ± 3.1	2.5 ± 2.8	2.8 ± 2.7
Headache frequency	Number of headaches per month, average number ± SD	18.4 ± 9.4	18.2 ± 9.6	19.5 ± 8.7	17.7 ± 10.1
Headache severity	Average score ± SD	3.2 ± 0.6	3.2 ± 0.6	3.1 ± 0.6	3.1 ± 0.7
RADS	Total score (anxiety and depression), average ± SD	14.8 ± 8.3	15.9 ± 8.5	13.5 ± 8.3	14.9 ± 8.0
	Total anxiety score, average ± SD	7.7 ± 4.8	8.4 ± 5.0	7.0 ± 4.8	7.8 ± 4.7
	Total depression score, Average ± SD	6.4 *±* 3.6	6.9 ± 3.8	5.8 ± 3.6	6.5 ± 3.5
PCQ	Average score ± SD	99.6 ± 17.4	95.6 ± 14.2	102.1 ± 19.9	101.4 ± 17.5
CSI	Average score ± SD	20.7 ± 14.1	22.5 ± 12.4	19.7 ± 16.6	19.6 ± 13.3

Full analysis set of all randomised children into the study

### Primary outcome measures

In Table 2, the results of the primary outcome measures are represented, based on the ITT analysis. Headache frequency, the primary outcome, was significantly reduced in all groups (from 18.9 days to 12.5 and 10.5 at respectively 3 and 9 months; *p* < 0.001) with no significant differences observed between the groups at both 3-month and 9-month follow-ups. The percentage of children with a clinically relevant reduction in headache frequency was 41% in total at 3 months and 47% at 9 months with no significant differences between the groups. Similar results were found in the PP analysis (data not shown).

**Table 2:** Primary and secondary outcome measures after 3 and 9 months of intervention: ITT analyses

Outcome variable	All (N = 123)	HT (N = 45)	TM (N = 37)	PMR (N = 41)	Difference versus baseline (*p*)	Difference between interventions (*p*)
Number of days with headache per 4 weeks (mean ± SD)						
Baseline	18.9 (9.5)	18.2 (9.6)	20.1 (9.0)	18.5 (9.8)	–	0.64^b^
3 months	12.5 (8.8)	12.1 (9.4)	13.2 (9.6)	12.3 (7.6)	*< 0.001* ^a^	0.85^b^
9 months	10.5 (9.0)	9.8 (9.8)	11.8 (9.1)	10.0 (7.9)	*< 0.001* ^a^	0.57^b^
≥ 50% reduction in headache frequency (%)						
Baseline	–	–	–	–	–	–
3 months	50 (41%)	20 (44%)	15 (41%)	15 (37%)	–	0.76^c^
9 months	58 (47%)	24 (53%)	17 (46%)	17 (42%)	0.17^d^	0.54^c^

^a^Paired *t* test

^b^One-sided ANOVA

^c^Chi-squared test

^d^McNemar test

Italicized *p*-values indicate a statistically significant difference

### Secondary outcome measures

The secondary outcome measures (ITT analyses) are presented in Table 3: 32% and 43% of the children reported adequate relief at 3 and 9 months respectively, with no significant difference between the intervention groups. Depression scores were reduced at 9 months (*p* = 0.04), with no difference between the intervention groups. Coping with pain scores showed no significant changes after the intervention period for all groups. In the Children Somatisation Inventory, there was a significant decrease after both 3 (*p* = 0.001) and 9 months (*p* < 0.001), again without significant differences between the groups. Similar results were found in the PP analysis. No adverse events were reported during the different interventions.

**Table 3:** Secondary outcome measures after 3 and 9 months of intervention: ITT analysis

Outcome variable	All (N = 123)	HT (N = 45)	TM (N = 37)	PMR (N = 41)	Difference versus baseline (*p*)	Difference between interventions (*p*)^e^
Adequate relief (%)						
Baseline	*–*	*–*	*–*	*–*	–	–
3 months	39 (32%)	19 (42%)	10 (27%)	10 (24%)	–	0.16^c^
9 months	53 (43%)	24 (53%)	13 (35%)	16 (39%)	*< 0.001* ^d^	0.21^c^
RCADS (total) (mean ± SD)						
Baseline	14.5 (8.3)	15.9 (8.5)	12.5 (7.9)	14.7 (8.2)	–	0.12^b^
3 months	13.8 (9.4)	14.9 (10.1)	12.4 (9.6)	13.8 (8.5)	0.53^a^	0.11^b^
9 months	12.4 (9.0)	13.5 (10.0)	11.0 (8.2)	12.3 (8.5)	0.06^a^	0.16^b^
RCADS (anxiety) (mean ± SD)						
Baseline	7.6 (4.8)	8.4 (5.0)	6.4 (4.5)	7.6 (4.8)	–	0.28^b^
3 months	7.4 (5.4)	8.0 (5.6)	6.5 (5.4)	7.6 (5.2)	0.80^a^	0.24^b^
9 months	6.5 (5.1)	6.9 (5.5)	5.8 (4.8)	6.8 (5.1)	0.11^a^	0.31^b^
RCADS (depression) (mean ± SD)						
Baseline	6.3 (3.6)	6.9 (3.8)	5.5 (3.5)	6.4 (3.6)	–	0.07^b^
3 months	5.8 (3,9)	6.4 (4.5)	5.2 (3.8)	5.7 (3.4)	0.30^a^	0.16^b^
9 months	5.3 (3,8)	6.0 (4.3)	4.8 (3.3)	5.0 (3.4)	*0.04* ^a^	0.15^b^
PCQ (mean ± SD)						
Baseline	99.4 (17.8)	95.5 (14.2)	101.5 (21.1)	101,8 (17.8)	–	0.19^b^
3 months	98.1 (19.9)	97.0 (19.6)	97.3 (21.0)	100.1 (19.6)	0.32^a^	0.75^b^
9 months	97.7 (19.2)	96.4 (20.4)	97.6 (18.8)	99.3 (18.4)	0.27^a^	0.78^b^
CSI (mean ± SD)						
Baseline	20.3 (13.7)	22.5 (12.4)	18.4 (15.3)	19.6 (13.5)	–	0.36^b^
3 months	17.3 (13.6)	17.9 (13.0)	17.3 (15.2)	16.7 (12.9)	*0.001* ^a^	0.92^b^
9 months	15.6 (12.2)	16.0 (12.3)	15.5 (13.2)	15.2 (11.5)	*< 0.001* ^a^	0.94^b^

^a^Paired *t* test

^b^One-sided ANOVA

^c^Chi-squared test

^d^McNemar test

^e^*p* values were corrected for baseline differences between the groups if these were present

Italicized *p*-values indicate a statistically significant difference

### Additional analyses

No correlations were found between the absolute headache frequency differences at 3 and 9 months and the baseline scores for RCADS, CSI and PCQ or the number of times that the children applied the intervention at home (results not shown). Similar results were found in the PP dataset.

Sub-group analysis showed an overall difference in the absolute headache frequency difference between boys and girls (*p* = 0.009), girls responding better to the intervention than boys. This difference was not found in the PP analysis.

In the ITT analyses, children with TTH responded to treatment whereas children with migraine did not (differences at 9 months versus baseline: TTH; 10.1 ± 8.8 versus migraine; − 0.8 ± 5.2 days with headache, *p* < 0.001). Similar results were found in the PP dataset. However, after adjustments for baseline headache frequency, the differences in response between TTH versus migraine were not significant anymore (*p* = 0.079).

Thirty-five children received the treatment that they preferred (HT: *N* = 20, TM: *N* = 10, PMR: *N* = 5), and 26 children did not receive the preferred treatment (HT: *N* = 4, TM: *N* = 6, PMR: *N* = 16). The remaining parents/children had no treatment preference. No correlations were found between outcomes and treatment preferences (data not shown).

Use of pain medication was significantly reduced in all children at 3 months (*p* = 0.003) and 9 months (*p* = 0.032), but no significant differences were observed between the three groups. There were few children that used medical prophylactic treatment during the course of the study: *N* = 2 in the HT group, *N* = 3 in the TM group and *N* = 3 in the PMR group.

## Discussion

This is the first RCT that has examined the comparative effectiveness of mind-body techniques in children with primary headaches. All three techniques that were studied, i.e. HT, TM and PMR exercises, effectively reduced primary headaches with a reduction of > 50% of headache days in 41% of the children directly after treatment, and in 47% at 9-month follow-up, without significant differences between the groups. A systematic review on the placebo effects in headache trials reported an overall clinically relevant improvement in 14.1% of the children in no treatment control groups of non-pharmacological trials [6], suggesting that the observed effects of HT, TM and PMR with recovery rates ranging between 37 and 44%, are, for a large part, specific treatment effects.

Previous studies have demonstrated the effectiveness of HT in children with functional abdominal complaints and irritable bowel syndrome, with adequate relief in more than 80% of the children at 1-year follow-up [27, 34]. The relatively high treatment success of HT in children with abdominal pain, compared to the results in this study in children with primary headache, may possibly be explained by different characteristics of these two types of chronic pain. It has been reported that children with chronic abdominal pain have higher levels of anxiety and depression than children with primary headache [38]. Hence, the mechanisms by which HT may exert its effect may, to a large extent, be related through reduction of these other pain and stress-related symptoms. However, we did not find a relation between anxiety and depression scores at baseline and response to therapy; therefore, other differences may exist between these groups of children with chronic pain that might explain the difference in response to HT.

It is not possible to compare results from the present study in the TM group with previous studies, since this is the first study that examined the effect of a meditation technique in children with primary headaches. Three small studies in adults, using different meditation techniques, showed positive effects on pain intensity and quality of life in comparison to control groups [1, 13, 36].

Since this study suffered from several limitations, findings should be interpreted with caution. The study was powered to demonstrate a possible difference between the HT or TM intervention versus a control group receiving standard medical treatment. However, it became apparent that in some participating hospitals, children with primary headache were referred to PMR exercises by a physiotherapist or psychologist as part of standard treatment. It was therefore decided to follow an “active” control group in all six hospitals, i.e. standard medical treatment plus PMR exercises. The active control group in the present study should thus be regarded as an intervention group and did not serve its purpose as a control group as was intended in the sample size calculation. If such a control group might have been included, demonstrating a similar recovery rate in 12% of children as reported by Larsson et al. [15], the observed recovery rates of 53% in the HT and 46% in the TM group might have been significantly better. Four years were needed in order to recruit the estimated number of children needed in the study. The children that chose to participate in the study might therefore not be a representative sample. It turned out that on average, only one third of the children consulting the participating hospitals for primary headache was willing to participate in the trial. Main reasons for declining participation were the expected time investment for children and parents, or that the randomly selected intervention would not fit the child. Also, the travel distance to the therapists played a role whether or not to participate. Furthermore, the drop-out rate in this study was larger than 20% in the TM and PMR group. The main reasons for drop-out were that the therapy did not fit the child and the long travel distance to the place of the specific intervention. Future studies, therefore, should consider whether it is feasible to provide the relaxation interventions at the site of the hospital itself. The results of the present study indicate that as part of an integrative primary headache treatment plan, parents and/or children can choose either one of the investigated techniques, depending on personal preferences and those offered at the hospital, in line with the practical recommendations for stress management in paediatric headaches as recently published [3]. All three interventions studied appeared to be safe, since no adverse events were reported.

In conclusion, non-pharmacological techniques added to standard medical care effectively reduce the number of primary headache in children without adverse events. This study shows comparable effectiveness for hypnotherapy, transcendental meditation or progressive muscle relaxation exercises provided by a physiotherapist or psychologist.

## Abbreviations

CWZ: Canisius-Wilhelmina Ziekenhuis
CBT: Cognitive behavioural therapy
CSI: Children Somatisation Inventory
FAS: Full analysis set
HT: Hypnotherapy
ITT: Intention to treat
JKZ: Juliana Kinderziekenhuis
LOCF: Last observation carried forward
MMC: Maxima Medisch Centrum
MRI: Magnetic resonance imaging
PP: Per-protocol
PCQ: Pain Coping Questionnaire
PMR: Progressive muscle relaxation
RCADS: Revised Children’s Anxiety and Depression Scale
RCTs: Randomized clinical trials
SD: Standard deviation
TM: Transcendental meditation
TTH: Tension-type headache
